# Supplementary material for: Are carbon emissions trading and green financial instruments synergistic? -Comprehensive quantitative research based on content analysis
Source: PLoS One. 2024 Mar 7;19(3):e0298601. doi: 10.1371/journal.pone.0298601 (PMC10919601; doi:10.1371/journal.pone.0298601)
Supplement: S1 Data — (ZIP) [file pone.0298601.s001.zip › Data/地级市Z_F工作报告.zip!/╣π╢1⁄2/╜¡├┼╩╨/└┤╫╘╣1⁄2╓┌║┼í╛┬φ┐╦╩2╛▌═°í┐_2009.docx]

我代表市人民政府向大会报告政府工作，请予审议，并请市政协各位委员和其他列席人员提出意见。

　　2008年工作回顾

　　在极不平凡的过去一年中，我们深入贯彻落实科学发展观，继续解放思想，坚持改革开放，积极应对国际金融危机的冲击，全市经济保持较快发展，民生持续改善，社会和谐稳定，基本完成了市十三届人大三次会议确定的各项目标任务。

　　——过去的一年是积极应对国内外复杂形势，经济保持平稳较快发展的一年。

　　面对急剧变化的国际国内经济形势，我们迅速应对，积极贯彻落实中央、省扩大内需政策，推动经济平稳较快发展。全市完成生产总值1280.6亿元，增长10.8%，高于全省平均增速。人均生产总值达30973元，增长10.4%。地方财政一般预算收入在减收因素增多的情况下仍然保持较快增长，达到74.7亿元，增长19.6%。外贸进出口总额131.6亿美元，增长7%，其中出口总额96.7亿美元，增长11.2%；实际吸收外商直接投资9.2亿美元，增长17%。全社会固定资产投资386.7亿元，增长19.2%。社会消费品零售总额489.3亿元，增长19.5%。居民消费价格总水平上涨4.5%，低于全国、全省平均涨幅。经济发展质量进一步提升，地方财政一般预算收入占生产总值的比重由上年的5.6%提高到5.8%，高新技术产品产值占工业总产值的比重由上年的30%提高到32%。

　　——过去的一年是建设现代产业体系，产业结构调整优化的一年。

　　“四大百亿工程”取得新成效，全市实现规模以上工业总产值2751.2亿元，增长20%；增加值640.7亿元，增长16.3%；产值超100亿元的镇（街）发展到8个，电子信息、交通运输设备、化纤纺织服装、金属制品等4个产业集群产值超300亿元，船舶制造、绿色光源、石化、造纸等产业实现强势增长。自主创新能力提升，高新技术企业增至201家，新增2个国家级产业基地、1个省级专业镇技术创新平台、16个省市级工程中心和3个中国驰名商标、6个省名牌产品、9个省著名商标，信息化综合指数由上年的76.1%提高到77.8%。旅游业实现总收入90.6亿元，增长30.2%；旅游外汇收入3.9亿美元，增长116.5%。发展和利用资本市场取得新进展，2家企业进入上市辅导期。交通银行江门分行开业，恩平汇丰村镇银行试业。节约集约发展取得新成效，单位生产总值能耗下降5.2%。淘汰落后产能步伐加快，共关停小火电21.5万千瓦、小钢铁95万吨、小水泥100万吨。共清理闲置土地4.6万亩，工业项目土地投资强度比上年提高37.7%，违法违规用地得到有效整改。

　　——过去的一年是加大“三农”工作力度建设扎实推进的一年。

　　全市各级财政“三农”投入28.5亿元，增长19.9%。成功举办首届农业博览会，签订农业合作项目112个，总投资额达31亿元。农业产业化经营取得新进展，新增10家市级以上农业龙头企业、22家农民专业合作经济组织。农业产业化组织带动32万多农户，占全市农户数的41.1%，户均增收1490元。镇级经济快速发展，镇级地方财政一般预算收入34.5亿元，增长26.6%，财政收入超千万元和超亿元的镇（街）分别增至61个和6个。海洋产业规模壮大，实现产值151.5亿元，增长11%。现代林业加快发展，完成营造林22.9万亩。农业农村基础设施建设力度加大，省人大小型水库除险加固和水库移民议案全部完成，城乡水利防灾减灾工程稳步推进；完成农田整治6.6万亩，无公害农产品生产基地发展到100.2万亩；镇通行政村公路硬底化全面实现。村庄整治扎实推进。“千企扶千村”就业工程成效显著，全市1050个村委会与1296家企业签订了劳务合作协议书，免费培训农村劳动力4.3万人，向二、三产业转移农村富余劳动力3.8万人；全市农村居民人均工资性收入达4050元，比上年增加395元，增长10.8%。华侨农场改革发展取得新成绩。

　　——过去的一年是加大基础设施投入，环境保护和城乡建设取得新进展的一年。

　　全市110项重点项目完成投资97.4亿元，总投资16.1亿元的18个项目纳入国家扩大内需计划。完成市区公共交通总体规划、江门港总体规划及其环境影响评价的编制。滨江新区启动区建设加快推进。广珠轻轨江门支线、广珠铁路江门段、佛开高速公路扩建工程和川岛风电首期工程进展顺利。江肇高速公路和500千伏五邑输变电工程动工。台山核电、台山电厂二期工程前期工作进展顺利。生态环境保护力度加大，共否决、劝退高能耗、重污染类规模以上项目17个。大力实施减排工程，完成减排项目81个。新建成4家污水处理厂，全市城镇生活污水处理率由上年的33.6%提高到50%以上。二氧化硫和化学需氧量分别削减4%和3.5%。全市环境质量继续保持较好水平，大气环境质量维持在国家二级标准，市区空气质量日报优良率96.7%；城市水域功能区和饮用水源水质达标率100%；森林覆盖率达41.4%。

　　——过去的一年是着力改善民生，社会各项事业全面发展的一年。

　　各级财政民生投入37.3亿元，增长35.1%。实施城乡居民收入倍增计划，城镇居民人均可支配收入17196元，增长13.5%，创近13年来新高；农村居民人均纯收入6807元，增长11.5%，创近10年来新高；城乡居民储蓄存款余额达1158.2亿元，增长19%。

　　新增就业岗位5.3万个，安置城镇劳动力和下岗失业人员再就业4.8万人，援助就业困难人员7900多人，城镇登记失业率控制在2.6%以内。进一步健全企业工资支付监控机制。提高基本养老保险待遇，全市参加养老保险人数比上年增长14.4%。建成廉租房449套，新增685户廉租房保障家庭。加大教育强市建设力度，全市各级财政教育投入18.7亿元，增长34%。

　　全面实现城乡免费义务教育，全面落实中等职业学校国家助学金政策，进一步提高农村困难家庭义务教育阶段学生生活费补助标准。新创建2个教育强区、10个教育强镇（街）、7所国家级示范性普通高中。五邑大学顺利通过本科教学水平评估，江门职业技术学院和江门电视大学办学有新进步。农村中小学规范化建设和布局调整取得新进展。职业技术教育规模继续扩大。

　　群众性精神文明活动蓬勃开展，成为“创建全国文明城市工作先进市”。开展“爱在江门”创业人物、“双百”优秀务工人员、“十大敬老之星”和“十大模范市民”评选活动，大力推动义工工作，文化服务百镇千村行、侨乡书香节和社区文化建设成效显著。成功举办第三届华人嘉年华活动和首届世界江门青年大会。新增6个国家级非物质文化遗产、3个中国民间文化艺术之乡。市新青少年宫投入使用。

　　城乡医疗卫生服务体系进一步健全，全市各级财政公共卫生投入4.1亿元，增长44.4%。社区卫生服务和镇卫生院改革试点稳步推进。城镇居民基本医疗保险试点申请参保人数达46.8万人。农村合作医疗参保人数增至233.7万人，参合率达97.3%。中医药名市建设深入推进。

　　体育事业取得历史性突破，我市体育健儿李珊珊、李宏利和李汉华在北京奥运会和残奥会上勇夺两金两银一铜，全民健身活动蓬勃开展。

　　市区全面实现免费婚检，低生育水平进一步稳定，完成省下达的人口计生工作任务。国防建设和民兵预备役工作进一步加强，实现“广东省双拥模范城”六连冠。残疾人工作上新水平。社会治安和安全生产形势保持稳定。

　　“平安江门”建设稳步推进，“六好”平安和谐社区由上年的43个增至106个。认真落实信访条例，一大批涉及群众切身利益的问题得到有效解决。监狱劳教、社区矫正工作得到加强。

　　产品质量和食品安全监管整治取得实效。安全生产“隐患治理年”活动成效显著，重特大事故得到有效遏制。完善应急预案体系，应急处置能力得到提高。侨务、外事、对台、民族、宗教、老龄、人防、统计、审计、档案、新闻、出版、方志、气象、红十字会等工作取得新成绩。

　　——过去的一年是增强政府执行力，政府服务水平进一步提高的一年。

　　积极应对严重雨雪冰冻和“黑格比”超强等自然灾害，安排财政专项贴息资金帮助受灾群众恢复生产。举全市之力支援地震灾区抗震救灾和对口援建，全市各界捐款捐物共计1.5亿元，接收复课师生近1500名；市财政拨付6243万元资金支援四川汶川县雁门乡恢复重建工作，已开工建设项目18项，援建工作走在全省前列。

　　积极应对国际金融危机的冲击，及时出台扩大内需、企业减负、加强政银企合作的政策措施，在市直机关开展与企业和困难群众“同心携手、共渡难关”活动，有力促进了经济平稳发展。

　　认真执行市人大及其常委会各项决议并向其报告工作，坚持向人民政协通报情况，自觉接受监督。共办理人大代表建议130件、政协提案137件，满意基本满意率分别达98.1%和100%。密切与工会、共青团、妇联等群众团体的联系，广泛听取社会各界意见。

　　村民居民自治制度不断健全。村（居）两委换届顺利完成。政务、厂务、村务公开工作取得新成果。推进综合行政执法体制、市区义务教育阶段办学体制和公安管理改革。

　　深化财政国库管理和政府采购制度改革，理财水平有新提高。

　　实现“110”、“119”、“120”、“122”应急求助电话号码“四合一”，建成市电子公文交换系统、行政审批电子监察系统和网上行政服务系统三期项目，开通“企政通”系统，搭建企业与政府实时互动信息平台。市行政服务中心和“12345”政府服务热线服务满意度分别达99%和98%。

　　在全省率先推行服务对象直接评议机关，并把群众满意度作为检验机关作风建设成效的主要标准；推行首办（首问）责任制，试行热点部门公开和落实“责任白皮书”，持续推进ISO质量管理体系有效运行，市直机关作风建设满意年活动初见成效。效能监察、廉政建设和反腐败工作进一步加强，一批违纪违法案件得到查处。

　　各位代表！过去的一年，我市在复杂多变的国内外形势和历史罕见的重大挑战面前，经济社会发展能取得这样的成绩，确实来之不易。这是在省委、省政府和市委的正确领导下，全市人民团结奋斗的结果，凝聚着社会各方面的心血和智慧。在此，我代表市人民政府，向全市广大工人、农民、知识分子、干部职工，向驻我市人民解放军、武警官兵，人民警察和各民主党派、人民团体、社会各界人士，致以崇高的敬意！向长期关心支持我市发展的港澳台同胞、海外侨胞以及国际友人表示衷心的感谢！

　　我们也清醒认识到，我市经济社会发展中还存在着产业结构不合理、竞争力不强，粗放型经济发展方式还未有根本性转变，服务业比重偏低，社会事业发展滞后等不少突出问题。受国际金融危机影响，我市还面临一系列新问题，主要有：财政减收增支因素增多；招商引资和外经贸形势严峻；部分企业生产经营困难加剧；保障和改善民生的任务十分艰巨；就业压力加大，影响社会稳定的因素增多；政府服务质量和效率与群众、企业和基层的期望仍有较大差距。对此，我们必须高度重视，立足当前，着眼长远，认真采取措施逐步加以解决。

　　2009年工作任务

　　今年经济社会发展预期目标

　　生产总值增长10.5%，力争人均生产总值与生产总值同步增长。

　　单位生产总值能耗下降4%，二氧化硫和化学需氧量分别削减6%和2%。

　　全社会固定资产投资增长16%。规模以上工业增加值增长14%。

　　外贸进出口总额增长8%。

　　地方财政一般预算收入增长10%。

　　社会消费品零售总额增长15%，居民消费价格总水平涨幅控制在4%以内。

　　城镇登记失业率控制在4%以内。

　　人口自然增长率控制在6.8％。以内。

　　城镇居民人均可支配收入增长9%，农村居民人均纯收入增长8%。

　　2009年是新中国成立60周年，是贯彻落实中央扩大内需政策，实现规划目标的关键一年，也将是进入新世纪以来经济发展最为困难的一年，同时也是实现“弯道超车”的机遇之年。今年市政府工作的总体要求是：

　　高举中国特色社会主义伟大旗帜，以党的十七大、十七届三中全会、中央经济工作会议、省委十届四次全会和市委十一届五次全会精神为指导，深入贯彻落实科学发展观，继续解放思想，坚持改革开放，抓住《珠江三角洲地区改革发展规划纲要》实施机遇，把“三促进一保持”作为抓发展的工作重点，着力落实中央、省扩大内需政策，加强基础设施建设，推进发展方式转变和结构调整，加快建设现代产业体系，加大招商引资工作力度，深化农村改革发展，突出保障和改善民生，促进经济社会又好又快发展。

　　今年经济社会发展的预期目标是：

　　生产总值增长10.5%，力争人均生产总值与生产总值同步增长，单位生产总值能耗下降4%，二氧化硫和化学需氧量分别削减6%和2%，全社会固定资产投资增长16%，规模以上工业增加值增长14%，外贸进出口总额增长8%，地方财政一般预算收入增长10%，社会消费品零售总额增长15%，居民消费价格总水平涨幅控制在4%以内，城镇登记失业率控制在4%以内，人口自然增长率控制在6.8％。以内，城镇居民人均可支配收入增长9%，农村居民人均纯收入增长8%。

　　为实现上述目标，今年市政府要重点推进以下工作：

　　一、加大力度推进投资和消费，千方百计扩大内需

　　进一步加大投资力度。今年投资283亿元建设137项市重点项目，带动全社会固定资产投资450亿元。积极创造条件，争取国家和省批准一批、提前开工建设一批、提早建成一批、积极储备一批重大项目。全力配合广珠轻轨江门支线、广珠铁路江门段、南沙铁路、江肇高速等工程建设，争取广佛江珠轻轨、西部沿海铁路、江罗高速、中开高速、崖门水道和崖门出海航道全潮1万吨乘潮3万吨等项目提前动工。加快新会港二期、江顺大桥、东华大桥、大洞大桥、礼东大桥、鹤南西江大桥等22个重点交通基础设施项目，台山核电、台山电厂二期、江门输变电工程、新会双水发电厂热电联供机组等7个能源项目，信达高分子纤维、信义玻璃、丽得LED、富华重工、艾默生精密空调等30个重大产业项目，以及旧城改造和城中村改造等城建项目建设。加大城乡水利防灾减灾等工程投资力度。

　　积极扩大消费需求。完善促进就业、强农惠农机制，促进城乡居民增加收入。落实“家电下乡”消费政策，深入实施“万村千乡”市场工程，扩大农村地区消费。推行市民旅游休闲计划，打造休闲旅游黄金线路。稳定和扩大房地产、汽车消费，激发文化、体育、健康消费活力，培育新的消费热点。整顿和规范市场经济秩序，加强对涉及人民群众身体健康和生命安全的食品药品等重点产品的监管，保障消费者合法权益，为城乡居民营造安全放心的消费环境。

　　二、加大力度推进《纲要》实施，增创科学发展新优势

　　制定贯彻落实《珠江三角洲地区改革发展规划纲要》的工作方案，在“科学发展、先行先试”上实现新突破。

　　努力在建设探索科学发展模式试验区上先行先试，以新会区为试点率先探索经济发展方式转变、城乡区域协调发展、和谐社会建设的新途径和新举措。

　　在实施CEPA、深化与港澳及珠三角核心城市合作上先行先试，以互利为核心，以共建为机制，以利益共同体为载体，在交通、环保、旅游和产业等方面实现与包括港澳在内的珠三角都市群的实质性合作。

　　在解决产业同构化上先行先试，制定产业发展布局规划，通过经济手段和行政手段双管齐下，打破行政区域界限，推动产业集聚发展。

　　在建设节约集约用地示范试点上先行先试，推进山坡地、滩涂以及围海造地开发利用。

　　在区域协调发展上先行先试，深化山海合作和异地发展，精心做好江门产业转移工业园的申报工作，争取成为省级产业转移工业园和省示范性产业转移工业园。

　　在城乡统筹发展上先行先试，建立城乡统筹的社会保障体系；推进“千企扶千村”就业工程，年内培训农村青年和农村富余劳动力4万人，实现转移就业3万人，努力建设全省重要的职业技术教育基地、一流的职业技能开发评价示范基地和创业带动就业孵化基地。

　　在农村信息化建设上先行先试，以村村通互联网为载体，建立农业科技、流通、农民就业、计生、文化、教育服务平台，在全部农业镇、50%以上的行政村和农民专业合作经济组织建立信息化服务站点。

　　在建设高水平、高品质社会事业发展示范区上先行先试，争创国家教育综合改革示范区、公共文化建设示范区和医药卫生体制改革试验区。

　　在行政管理改革上先行先试，遵循精简、统一、效能的改革方向，进一步优化政府组织体系和运行机制。

　　在打造服务型政府上先行先试，努力建设人民满意政府。

　　三、加大力度推进现代产业体系建设，增强产业竞争力

　　大力打造先进制造业基地。加快汽车零部件、绿色（半导体）光源、造纸等产业基地建设；做大做强摩托车及汽车零部件、石化中下游、船舶制造、食品、电子信息等产业集群；着力抓好与省共建国家电子信息制造业基地工作。市级投入9380万元加大对企业自主创新和技改项目的扶持力度，择优挑选100家有品牌、有市场、自主创新能力强的企业给予重点扶持，推动35家以上传统企业转型升级。积极引导企业用好增值税转型和企业研发费用税前加计扣除等自主创新优惠政策，加大技术改造投资力度。

　　积极发展现代服务业。把物流业和旅游业作为现代服务业发展的重点。推进新会银洲湖、鹤山（沙坪—桃源）和南新区等现代物流基地建设，争取在建设大型物流基地和专业市场上有新突破。进一步提升旅游资源的营销和整合水平，加强与港澳台、周边省区及国际旅游合作，打响温泉、海滨“三张牌”，保持旅游业强势增长。加快建设蓬江区中央商务区，大力推进核心城区“退二进三”战略。壮大文化、信息等新兴服务业，合理布局便民利民的传统服务业。

　　促进中小企业加速成长。市级财政投入2180万元专项资金支持中小企业发展。加快实施民营及中小企业成长工程，鼓励民营企业创立品牌、创新技术、规范管理。每月举办一次市级政银企信息交流会，促进银企实质性合作。鼓励设立各类信贷公司，探索建立中小企业担保基金和区域性再担保机构，安排专项资金以创业补助、业绩奖励或风险补偿等方式扶持担保公司发展。继续扶持企业上市融资。深化投资体制改革，改善投资监督管理方式。抓好国有企业改制重组，鼓励民资进入市政公用事业领域。

　　加快提升自主创新能力。加大自主创新投入，努力提高研发经费支出占生产总值的比重。新培育25家以上高新技术企业，力争全市高新技术产品产值占工业总产值的比重达到34%。加快企业工程技术研发中心建设，力争新增省市级工程技术研发中心10家。加快推进国家级摩托车质量检测中心、绿色光源检测中心和各类科技成果孵化器建设。积极引导行业龙头骨干企业与高校合作，组织实施产学研合作项目50项、省市级科技计划

　　300项，推广科技成果200项以上，力争专利申请量超3000件。大力实施专业镇技术创新试点工程。实施名标名牌带动战略，推动产业集群创建区域品牌。完善知识产权管理服务体系，加强知识产权保护和专利行政执法。支持企业参与行业、国家和国际标准的制定。创新人才聚集的体制机制，设置“江门杰出人才奖”，积极引进高层次人才。

　　提高开放型经济水平。落实各项外贸扶持政策，积极调整出口市场结构和产品结构，引导企业重点开拓拉美、非洲、东欧等新兴市场，构建更加多元、稳定的国际贸易格局。促进加工贸易转型升级，鼓励加工贸易企业扩大国内采购和产品内销。加快办理出口退税，重点支持自主品牌产品和有竞争力的劳动密集型产品扩大一般贸易出口。支持企业在研发、生产、销售等方面开展国际化经营。

　　四、加大力度推进招商引资，增强经济发展后劲

　　营造招商引资新洼地。落实《关于应对当前经济形势进一步减轻企业负担的意见》和《关于进一步加强招商引资工作的意见》，建立和完善招商引资领导机制、工作机制和考核制度。加大招商引资政策优惠力度，在重点工业园区实行“零收费”，规范涉企检查和处罚，打造投资成本低、服务效率高、客商满意的投资环境。

　　加快产业载体建设。着力抓好核心园区建设，加快市高新区“二次创业”步伐，争取成为国家级高新技术产业开发区；搞好银洲湖、广海湾、江沙工业走廊、开平翠山湖、恩平米仓等园区规划，提高开发建设水平。开展旧城区、旧厂房、旧村庄低效用地清查工作，采取土地置换等形式缓解招商用地矛盾。

　　提高招商引资水平。拓宽招商引资渠道，加强与各地商会、行业协会、台协、工商联的沟通联系，广泛挖掘社会招商资源，充分发挥招商顾问、中介组织的作用，实行驻点招商、小分队招商和网络招商。在积极引进外资项目特别是世界500强企业的同时，下大力气引进央属、省属企业和民资企业。抓好企业增资扩产，提高合同资金到位率，力争实际利用外资民资增长10%。

　　五、加大力度推进农村改革发展，激发农业农村发展活力

　　加强农村制度建设。稳定农村基本经营制度，加快农业经营方式转变。进一步完善农村土地确权、登记、颁证工作。加强土地承包经营权流转管理服务，积极探索建立土地承包经营权流转市场，按照依法自愿有偿原则发展多种形式的适度规模经营。完善征地补偿机制。继续深化集体林权制度改革。推进华侨农场改革和发展。

　　加大强农惠农力度。今年全市各级财政预算用于“三农”支出29.1亿元，落实各项强农惠农措施。大力发展特色、效益、生态农业，提高农业综合生产能力。投入2000万元启动现代农业综合示范基地建设。积极扶持发展专业大户、家庭农场、农民专业合作社等规模经营主体，新增4家市级以上农业龙头企业，组建和改造10家以上农民专业合作经济组织。充分发挥供销社和农民专业合作经济组织在农业流通中的作用。推行标准化生产和农产品质量认证。加强动植物疫病防控。开展政策性农业保险试点。积极引进多种形式的新型农村金融机构。建立政府扶持、多方参与、市场运作的农村信贷担保机制，大力扶持有条件的农民专业合作社开展信用合作。投入4.5亿元推进江新联围、台山深井水库和新会东方红水库加固等水利工程建设，鼓励农民开展小型农田水利设施综合整治。以建立健全被征地农民养老保险制度为重点，推进农村养老保险工作。

　　大力发展农村公共事业。强化行政村规划，突出整治农村“脏、乱、差”，建立“户集、村收、镇运、县（市）处理”的农村生活垃圾收集处理机制，探索投资少、见效快、运营省的镇村生活污水处理新途径，大力发展农村沼气，广泛创建生态文明村。抓紧解决农村饮水安全问题。

　　六、加大力度推进能源资源节约和环境保护，加快生态型宜居典范城市建设

　　着力抓好节能减排。严格执行节能问责制和“一票否决”制。完善节能减排统计、监测、考核体系建设。严格实行主要污染物排放总量控制制度，加快淘汰落后生产能力，确保按计划完成小火电、小钢铁、小水泥等关停任务。推行建筑节能和清洁生产。加快推进城镇生活污水处理厂、污水管网和工业污染源减排设施建设，确保文昌沙水质净化厂二期扩建、江海和恩平污水处理厂首期、潮连污水处理厂首期及截污管网工程建成并投入运行，全面完成中心镇污水处理设施建设，新增污水日处理能力20万吨以上。抓好公交行业推广应用清洁能源工作。探索建立主要污染物排放指标有偿使用制度，落实污染减排鼓励政策。确保242万亩生态公益林面积不减少。

　　着力抓好节约集约用地。完成土地利用总体规划修编和第二次土地调查。严格执行土地利用年度计划。开展土地储备工作，优化建设用地市场配置，确保重点项目和产业建设用地需求。加强国土所建设，提高土地执法监管水平，坚决遏制土地违法违规行为，巩固土地卫片执法检查整改成果。做好地质灾害防治工作。

　　着力推进滨江新区和市区一体化建设。完成滨江新区启动区控制性详细规划和专项规划编制。加快滨江新区配套基础设施以及滨江大道后续工程建设。完成启动区征地拆迁工作。抓好天沙河路、新南路（西段）、天沙河引水增流等工程规划建设。重点推进基础设施、产业布局、生态环保、城市规划建设一体化及合作机制创新，着力在文化场馆、供水、供气和公共交通等城市公共服务设施整合上实现突破，逐步实现市区无障碍对接。

　　七、加大力度推进社会事业发展，保障和改善民生

　　把改善民生摆在政府工作更加突出的位置，今年各级财政计划投入41亿元改善民生，比上年增长22.5%。

　　促进社会充分就业。安置城镇劳动力3万人。健全支持创业带动就业的服务体系，营造浓厚的全民创业氛围。探索人才市场管办分离。健全劳务招聘信息网络，加快推广远程招聘系统。坚持开展“充分就业社区”创建活动。积极做好高校毕业生就业工作。重点帮助低保家庭、残疾人和退役士兵就业，援助就业困难人员6000人。加快技师学院建设，继续抓好高技能人才培养。大力推行劳动合同制度和集体合同制度，强化劳动保障监察执法和劳动争议调解仲裁工作，切实维护企业和职工合法权益，发展和谐劳动关系。

　　健全社会保障体系。推进做实养老保险个人账户、社会保险扩面和地税全责征缴。做好城镇低收入家庭住房保障工作，重点抓好廉租房建设。完成华侨农场危房改造。加大社会救助力度，完善五保供养机制。做好优抚安置工作。大力发展居家养老和社会福利慈善事业。推进残疾人事业发展。

　　创建教育强市。全市各级财政预算教育投入20.4亿元。新创建2个教育强市（县）、21个教育强镇（街），建成广东省教育强市。推进义务教育均衡发展，加快普及高中阶段教育，大力发展职业技术教育和民办教育，鼓励与境外机构合作办学。提高五邑大学办学水平，做好江门职院教学迎评工作，启动江门电视大学扩建工程。加强农村中小学教师队伍建设，推进城乡教师交流。积极稳妥解决中小学代课教师问题，做好教师工资福利待遇“两相当”工作。

　　塑造文化品牌。用社会主义核心价值体系引领侨乡精神文明建设，推进创建全国文明城市工作。制定文化产业和文化品牌发展规划。抓好开平与村落等文化遗产的保护开发。推进经营性文化事业单位改革，培育文化产业骨干企业。搞好档案馆、文化馆、图书馆、博物馆和社区文化站建设，启动五邑华侨华人博物馆正式布展，年内新的江门广播电视中心投入使用。积极开展文化下乡、进社区活动，推进“农家书屋”和文化信息资源共享工程。加强文化市场管理。

　　提高医疗卫生服务水平。深化医药卫生体制改革，加强公共卫生和医疗服务体系建设，建立健全农村三级卫生服务网络和社区卫生服务中心，提高公共卫生服务资源利用效率。实现城镇居民医疗保险全覆盖，提高新型农村合作医疗管理和保障水平。完善疾病防控体系。加强卫生监督执法和药品监管体系建设。深入开展爱国卫生运动。大力推进中医药名市建设和民营医疗机构发展。

　　加强和改进社会管理。推进依法治市，加强普法工作，增强全民法治观念，依法保障公民权益，促进社会公平正义。鼓励企业积极主动承担社会责任。加强法律援助和司法救助。加快推进户籍制度改革，推动建立流动人口服务管理“一证通”制度。提高城市管理综合执法水平，加大“数字城管”推广力度。做好信访和社会稳定工作，加强社会矛盾纠纷排查调处和预警体系建设，有效预防和妥善处置群体性事件。推进平安和谐社区建设，力争全市“六好”平安和谐社区达到70%以上，启动农村社区建设试点工作。完善区域治安防控合作机制。依法严厉打击各类违法犯罪和暴力恐怖活动。做好监狱劳教和社区矫正工作。严格落实各级政府安全生产监管责任和企业安全生产主体责任，推动重点行业和领域隐患排查治理常态化，加强村（居）安监机构建设，依法严厉查处各类安全生产违法违规行为，有效遏制重特大事故发生。加快安全生产应急救援指挥中心和平台建设，提高应急预案管理和演练水平，组建市级危化应急救援队伍，增强应对突发事件能力。

　　扎实做好体育、人口计生等工作。加快全民健身设施网络建设，广泛开展群众性体育运动。提高竞技体育训练水平，积极迎战全运会。落实新的《广东省人口和计划生育条例》，突出做好流动人口和农村计划生育工作，提高出生人口素质。加强国防教育和国家安全教育，做好“双拥”和民兵预备役工作。加强新形势下依法管理宗教事务工作，发挥宗教界人士和信教群众在经济社会发展中的积极作用。继续做好侨务、外事、对台、老龄、人防、统计、审计、新闻、出版、方志、气象、红十字会等工作。

　　继续做好对口支援雁门乡灾后恢复重建工作。加快灾区农村居民住房、交通、水利、教育、卫生等援建项目建设，优先解决灾区群众基本生活问题。广泛动员社会各界参与，加强与灾区经贸、旅游、劳务等合作，积极利用市场手段促进灾区经济社会发展。

　　八、加大力度推进行政管理体制改革，建设服务型政府

　　深化行政管理体制改革。按照省批准我市的机构改革方案，推进市级和县级政府机构改革。深化行政审批制度改革，最大限度地减少和下放行政审批事项，扩大县级的管理权限。抓好新行政服务中心建设，实现市直、蓬江区、江海区所有审批部门开设窗口同址办公。加快新会区及各市行政服务中心建设。继续推进事业单位分类改革。

　　严格依法行政。认真落实人大及其常委会各项决议决定，坚持向人大及其常委会报告工作，自觉接受监督。主动加强与人民政协的联系并向其通报工作，支持政协委员发挥参政议政作用。提高议案、建议和提案办理质量。广泛听取各民主党派、工商联、无党派人士意见，支持工会、共青团和妇联等人民团体更好地发挥作用。发展基层民主，完善村居民自治制度。

　　提高理财治税水平。积极培育税源和新的税收增长点，提高税费征管的科学化、专业化和精细化水平，确保财政收入稳步增长。按市区一体化要求和事权财权相统一原则，完善市、区财政管理体制。按基本公共服务均等化的理财思路，从存量、结余中合理调整财政支出结构，保障基本公共服务支出。推进“镇财县（区）管”改革试点。严格控制一般性支出，勤俭办事，切实做到公务购车用车、会议经费、公务接待经费、出国（境）经费、办公经费预算“零增长”。

　　提升服务满意度。把服务对象满意度作为检验政府工作的标尺。健全政务公开、行政复议、投诉申诉等制度，畅通民意表达渠道，完善“12345”政府服务热线办理机制，扩大公众对政府工作的有序参与。加强电子政务建设，深入推进信息资源整合共享，拓展网上受理、并联审批和一站式服务。扎实推进ISO质量管理体系有效运行。建立服务对象满意度考核体系，完善公务员绩效激励机制。在落实首办（首问）责任制、否办退件把关制和涉企检查执罚把关知会制的同时，建立机关作风暗查制度，完善作风建设“一把手”负责制，实行“一把手”道歉制。继续开展机关作风建设年活动。深入开展与企业和困难群众“同心携手、共渡难关”活动。大力精简会议，压减文件数量和篇幅，大兴调查研究之风，把工作重心和主要精力放在落实“三促进一保持”的各项措施上。深入推进反腐倡廉建设，完善惩治和预防腐败体系。

　　各位代表！保持经济平稳较快发展，促进社会平安和谐，让全市人民增加幸福感，是市政府今年要努力实现的奋斗目标。让我们在省委、省政府和江门市委的正确领导下，高举中国特色社会主义伟大旗帜，深入贯彻落实科学发展观，解放思想，开拓创新，同心同德，扎实工作，共同谱写科学发展新篇章，以优异成绩迎接新中国成立60周年！
